# JNK‐mediated Ser27 phosphorylation and stabilization of SIRT1 promote growth and progression of colon cancer through deacetylation‐dependent activation of Snail

**DOI:** 10.1002/1878-0261.13143

**Published:** 2022-01-04

**Authors:** Yeon‐Hwa Lee, Su‐Jung Kim, Xizhu Fang, Na‐Young Song, Do‐Hee Kim, Jinyoung Suh, Hye‐Kyung Na, Kyung‐Ok Kim, Jeong‐Heum Baek, Young‐Joon Surh

**Affiliations:** ^1^ Research Institute of Pharmaceutical Sciences College of Pharmacy Seoul National University Seoul South Korea; ^2^ Department of Oral Biology Yonsei University College of Dentistry Seoul South Korea; ^3^ Department of Chemistry College of Convergence and Integrated Science Kyonggi University Suwon South Korea; ^4^ Department of Food Science and Biotechnology College of Knowledge‐Based Services Engineering Sungshin Women’s University Seoul South Korea; ^5^ Gachon Medical Research Institute Gil Medical Center Gachon University Incheon Korea; ^6^ Division of Colon and Rectal Surgery Department of Surgery Gil Medical Center Gachon University College of Medicine Incheon South Korea; ^7^ Department of Molecular Medicine and Biopharmaceutical Sciences Graduate School of Convergence Science and Technology Seoul National University Seoul South Korea; ^8^ Cancer Research Institute Seoul National University Seoul South Korea

**Keywords:** colon cancer, deacetylation, post‐translational modification, proinflammatory cytokines, SIRT1, snail

## Abstract

Sirtuin 1 (SIRT1), an NAD^+^‐dependent histone/protein deacetylase, has multifaceted functions in various biological events such as inflammation, aging, and energy metabolism. The role of SIRT1 in carcinogenesis, however, is still under debate. Recent studies have indicated that aberrant overexpression of SIRT1 is correlated with metastasis and poor prognosis in several types of malignancy, including colorectal cancer. In the present study, we found that both SIRT1 and SIRT1 phosphorylated on serine 27 were coordinately upregulated in colon cancer patients’ tissues and human colon cancer cell lines. This prompted us to investigate a role of phospho‐SIRT1 in the context of colon cancer progression. A phosphorylation‐defective mutant form of SIRT1, in which serine 27 was substituted by alanine (SIRT1‐S27A), exhibited lower protein stability compared to that of wild‐type SIRT1. Notably, human colon cancer (HCT‐116) cells harboring the SIRT1‐S27A mutation showed decreased cell proliferation and reduced capability to form xenograft tumor in athymic nude mice, which was accompanied by diminished transcriptional activity of Snail. HCT‐116 cells carrying SIRT1‐S27A were less capable of deacetylating the Snail protein, with a concomitant decrease in the levels of interleukin (IL)‐6 and IL‐8 mRNA transcripts. Taken together, these observations suggest that SIRT1 stabilized through phosphorylation on serine 27 exerts oncogenic effects at least partly through deacetylation‐dependent activation of Snail and subsequent transcription of IL‐6 and IL‐8 in human colon cancer cells.

## Graphical abstract

Overexpression of SIRT1 has been correlated to metastasis and poor prognosis; however, the role of SIRT1 in carcinogenesis is still under debate. Here, we report that both SIRT1 and P‐SIRT1^Ser27^ are upregulated in colon cancer patients and cell lines. Moreover, we observed that JNK catalyzed SIRT1 phosphorylation at Ser27, which led to deacetylation of Snail and its nuclear translocation. Consequently, the transcription of genes encoding proinflammatory cytokines was upregulated, which stimulated the growth and migration of colon cancer cells.

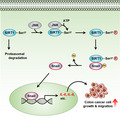

**Abbreviations**
CCL: C‐C motif chemokine ligand
CHX: cycloheximide
CRC: colorectal cancer
CXCL: C‐X‐C motif chemokine ligand
DMEM: Dulbecco's modified Eagle's medium
DYRK: dual‐specificity tyrosine phosphorylation‐regulated kinase
EMT: epithelial–mesenchymal transition
ERK: extracellular signal‐regulated kinase
H&E: hematoxylin and eosin
IHC: immunohistochemical
JNK: c‐Jun‐N‐terminal kinase

## 1 Introduction

Colorectal cancer (CRC), the third most common cancer in men and the second most commonly occurring cancer in women worldwide, is considered a global public health problem [1]. Besides genetic factors such as family history and inherited syndromes, inflammatory bowel disease may increase the incidence of CRC [2, 3, 4]. In addition, lifestyle factors, such as dietary habits and lack of physical activity, may also contribute to an increased risk of CRC [5, 6, 7]. Although combinations of surgical resection and therapies have been potentially applied to patients depending on the stage and the subtype of cancer, treatment options in this malignancy still remain suboptimal. Thus, gaining a better understanding of potential therapeutic target(s) of CRC may be needed to achieve a favorable clinical outcome.

Sirtuin 1 (SIRT1), an NAD^+^‐dependent class III histone deacetylase, is involved in aging, obesity, and metabolic diseases. Recent studies have shown that SIRT1 is overexpressed in CRC tissues as well as several colon cancer cell lines, suggesting that this enzyme may function as a tumor promoter [8, 9]. Moreover, SIRT1 overexpression is correlated with advanced stage and worse prognosis and hence predicts a poor overall survival in CRC patients [10, 11, 12].

Among a myriad of cellular substrates of SIRT1, p53 is the most well‐defined substrate to address the oncogenic function of SIRT1. Mechanistically, SIRT1 has been proposed to deacetylate p53 at the lysine 382 residue *in vitro* and *in vivo*, which may dampen its transcriptional activity [13]. In line with this proposition, knockdown of SIRT1 by use of a specific shRNA enhanced acetylation of p53 concomitantly with increased apoptosis in chronic myelogenous leukemia cells [14]. Likewise, pharmacological inhibition of SIRT1 induces apoptosis in human colon cancer cells (HCT‐116) and human glioma cell lines (U87MG and LN‐299) through acetylation‐dependent activation of p53 [15, 16]. Nevertheless, the molecular basis of regulation and oncogenic functions of SIRT1 in the progression of CRC has not been fully understood yet.

It has been proposed that phosphorylation of SIRT1 at the specific amino acids has unique effects on its protein stability [17, 18, 19] and catalytic activity [20, 21, 22]. Recently, hyperphosphorylation of SIRT1 has been observed in patients with CRC [23]. While an abnormality in the degree of phosphorylation of SIRT1 is likely to be implicated in the progression of some malignancies, its exact impact on SIRT1 itself and downstream signaling pathways has not been clearly elucidated.

Snail has been known to play a pivotal role in the epithelial–mesenchymal transition (EMT) during the embryonic development [24, 25] and late stage of tumorigenesis [26, 27, 28]. It binds to the E‐boxes in the *CDH1* promoter and represses transcription of the *CDH1* gene encoding an E‐cadherin, thereby boosting the EMT and acquisition of metastatic potential by tumor cells [26, 27, 28]. Even though the oncogenic function of Snail has been conventionally highlighted by transcriptional repression of the epithelial makers to accelerate the metastasis, accumulating evidence suggests that Snail is able to induce transcription of some oncogenic genes directly. For instance, Snail upregulates the expression of an actin‐dependent motor protein myosin Va involved in cell migration and metastasis of cancer, by binding to an E‐box1 at position −825 to −820 of the *MYO5A* promoter in HT‐29 human colon cancer cells [29]. Additional studies have also indicated that Snail is associated with transcriptional upregulation of several chemokines, including C‐X‐C motif chemokine ligand (CXCL) 1/2 and C‐C motif chemokine ligand (CCL) 2/5 and cytokines (TNF‐α, IL‐6, and IL‐8) [30, 31, 32].

Here, we report that phosphorylation of SIRT1 on the serine 27 residue by c‐Jun‐N‐terminal kinase (JNK) contributes to deacetylation‐dependent translocation of Snail to the nucleus and subsequent transcription of genes encoding proinflammatory cytokines IL‐6 and 8. This stimulates migration and growth of human colon cancer cells.

## 2 Materials and methods

### 2.1 Reagents

Dulbecco's modified Eagle's medium (DMEM) and FBS were purchased from Gibco‐BRL (by Thermo Fisher Scientific Inc.; Waltham, MA, USA). MG‐132 was a product of Enzo Life Sciences (Exeter, UK). Cycloheximide (CHX) and 3‐(4,5‐dimethylthiazol‐2‐yl)‐2,5‐diphenyltetrazolium bromide (MTT) were obtained from Sigma‐Aldrich (St. Louis, MO, USA). SP600125, U0126, and SB203580 were purchased from Tocris Bioscience (Bristol, UK). Matrigel® Growth Factor Reduced (GFR) Basement Membrane Matrix was supplied by CORNING Inc. (Corning, NY, USA).

### 2.2 Cell culture

Human colon cancer cell lines (HCT‐116, HCT‐15, DLD‐1, and SW480) were obtained from the Korean Cell Line Bank (KCLB), and normal human colon epithelial CCD841CoN cells were purchased from American Type Culture Collection (ATCC). All human cell lines have been authenticated using STR or SNP profiling within the last three years. HCT‐116 and SW480 cells were routinely maintained in DMEM, and HCT‐15 and DLD‐1 cells were grown in RPMI 1640. CCD841CoN cells were cultured in MEM containing 10% FBS and a 100 ng·mL^−1^ antibiotics mixture in a humidified atmosphere of 5% CO_2_/95% air. All experiments were performed with mycoplasma‐free cells.

### 2.3 Transient transfection with SIRT1 or Snail siRNA

Transient transfection of cells with siRNA (20 nm) targeting SIRT1 or Snail was carried out using Lipofectamine® RNAiMAX (Invitrogen by Thermo Fisher Scientific Inc.; Waltham, MA, USA) in accordance with the manufacturer’s procedure. Scrambled siRNA was used as a negative control. The target sequences for human SIRT1 siRNA #1 were 5′‐ACUUUGCUGUAACCCUGUA (dTdT)‐3′ (sense) and 5′‐UACAGGGUUACAGCAAAGU (dTdT)‐3′ (antisense); for human SIRT1 siRNA #2, 5′‐AGAGUUGCCACCCACACCU(dTdT)‐3′ (sense) and 5′‐AGGUGUGGGUGGCAACUCU(dTdT)‐3′(anti‐sense); and for human Snail siRNA, 5′‐GCGAGCUGCAGGACUCUAA(dTdT)‐3’ (sense) and 5′‐UUAGAGUCCUGCAGCUCGC(dTdT)‐3′ (antisense). siRNA oligonucleotides targeting SIRT1 or Snail were supplied by Genolution, Inc. (Seoul, South Korea). After 48 h of transfection, cells were harvested.

### 2.4 Transient transfection with wild type and phosphorylation‐defective plasmids

The FLAG tagged wild type SIRT1 (FLAG‐SIRT1‐WT) and mutant type SIRT1 in which serine 27 is substituted with alanine (FLAG‐SIRT1‐S27A) were custom synthesized by Cosmo Genetech (Seoul, South Korea). Transient transfection of cells with plasmid vectors was performed using Lipofectamine® 2000 (Invitrogen by Thermo Fisher Scientific Inc.; Waltham, MA, USA) as described previously [8]. After 48 h of transfection, cells were harvested or treated with MG‐132 or CHX according to the purpose of the experiment.

### 2.5 Generation of stable cell lines expressing SIRT1

pIRES‐SIRT1 was constructed by inserting DNA fragment of human SIRT1 resulting from cutting of the clone 1791 (Addgene; Watertown, MA, USA) into the pIRES vector, which contains the neomycin resistance gene. Subconfluent SW480 cells were transfected with pIRES or pIRES‐SIRT1 using Lipofectamine® 2000. After 48 h of transfection, cells were incubated with 0.8 mg·mL^−1^ of G‐418 (Roche; Mannheim, Germany) for a selection. Cells that survived the treatment with G‐418 were split into 96‐well plates at a density of 1 cell/well and cultured for several additional weeks in the presence of G‐418 for further selection. Single cells that grew into colonies were examined for constitutive expression of SIRT1 by Western blot analysis.

### 2.6 Western blot analysis

Cell lysates were subjected to 6–10% SDS/polyacrylamide gel followed by transfer to the polyvinylidene difluoride membranes as described previously [8, 33]. The membranes were then incubated with primary antibodies against SIRT1, Snail, actin (Santa Cruz Biotechnology, Inc.; Dallas, TX, USA); ubiquitin (Life Technologies by Thermo Fisher Scientific Inc.; Waltham, MA, USA); P‐SIRT1^Ser27^, P‐SIRT1^Ser47^, P‐JNK, JNK, acetylated‐lysine, and acetylated‐p53^Lys382^ (Cell Signaling Technology; Danvers, MA, USA). Blots were washed with Tris‐buffered saline with 0.1% Tween‐20 and were then probed with horseradish peroxidase‐conjugated secondary antibodies (Pierce Biotechnology; Rockford, IL, USA). The transferred proteins were detected by using Western blotting detection reagents (AbClon; Seoul, South Korea).

### 2.7 Reverse transcription‐polymerase chain reaction (RT‐PCR) and real‐time PCR (qPCR)

RT‐PCR and qPCR were carried out as described previously [8]. The primers used for the RT‐PCR are as follows (forward and reverse, respectively): for *SIRT1*, 5′‐GCAACATCTTATGATTGGCAC‐3′ (position 620 to 640) and 5′‐AAATACCATCCCTTGACCTGAA‐3′ (position 891 to 870) with a product size of 272 bp. For *IL‐6*, 5′‐GTGTGAAAGCAGCAAAGAGGC‐3′ (position 493 to 513) and 5′‐ CTGGAGGTACTCTAGGTATAC‐3′ (position 652 to 632) with a product size of 160 bp. For *IL‐8*, 5′‐ATGACTTCCAAGCTGGCCGTGGCT‐3′ (position 154 to 177) and 5′‐ TCTCAGCCCTCTTCAAAAACTTCT‐3′ (position 445 to 422) with a product size of 292 bp. For *Snail*, 5′‐CCTGCTGGCAGCCATCCCAC‐3′ (position 197 to 216) and 5′‐GGCAGCGTGTGGCTT CGGAT‐3’ (position 616 to 597) with a product size of 420 bp. For *CDH1*, 5′‐GCTGGAGATTAATCCGGACA‐3′ (position 1720 to 1739) and 5′‐ACCCACCTCTAAGGCCATCT‐3′ (position 2107 to 2088) with a product size of 388 bp. For *GAPDH*, 5′‐GCATGGCCTTCCGTGTCCCC‐3′ (position 857 to 876) and 5′‐ CAATGCCAGCCCCAGCGTCA‐3′ (position 1072 to 1053) with a product size of 216 bp. The primers used for the qPCR are as follows: for *SIRT1*, 5′‐AGAA GAACCCATGGAGGATG‐3' (position 1391 to 1410), and 5′‐TCATCTCCATCAGTCCCAAA‐3' (position 1504 to 1485) with a product size of 114 bp. For *RPL32*, 5′‐CTCTTTCCACGATGGCTTTG‐3' (position 437 to 418) and 5′‐GTCAAGGAGCTGGAAGTGCT‐3' (position 338 to 357) with a product size of 100 bp. PCR products were measured by 7500 Fast Real‐Time PCR System (Applied Biosystems by Thermo Fisher Scientific Inc.; Waltham, MA, USA) using fluorescent SYBR Green I dye.

### 2.8 Immunoprecipitation

Cell lysates were prepared as described previously and were immunoprecipitated with an appropriate antibody [34]. After resolving the samples by SDS/polyacrylamide gel electrophoresis, membranes were incubated with the indicated primary antibodies.

### 2.9 Wound healing assay

Cells were plated into culture‐insert (Ibidi; Martinsried, Germany) to create a 500‐μm gap uniformly. After removing the insert from a plate, cells were grown for an additional 48 h and then photographed under the microscope.

### 2.10 Clonogenic assay

Cells were seeded in 6‐well plates (200 cells·well^−1^) and grown for 14 days. Colonies were stained with 0.05% crystal violet. The results were determined by counting the number of colonies (more than 50 cells) manually under the microscope or by measuring the absorbance at 590 nm using a microplate reader following solubilization of crystal violet dye in 10% acetic acid.

### 2.11 Anchorage‐independent growth assay

Cells were prepared as described earlier [8]. Following incubation of cells in semisolid medium for 14 days, colonies were stained with 0.05% crystal violet. The number of colonies larger than 100 μm was counted under a microscope.

### 2.12 Invasion assay

Cells (4 × 10^4^) were added to the top chamber of the transwell (CORNING Inc., cat. no. 3422; Corning, NY, USA). DMEM supplemented with 10% FBS was added to the lower chamber. After 48‐h incubation, non‐invaded cells were removed by scrubbing with cotton swab. Cells on the lower surface of the membrane were fixed with 100% methanol for 2 min and stained with 0.05% crystal violet. The number of invaded cells was counted under a microscope.

**Fig. 1:**
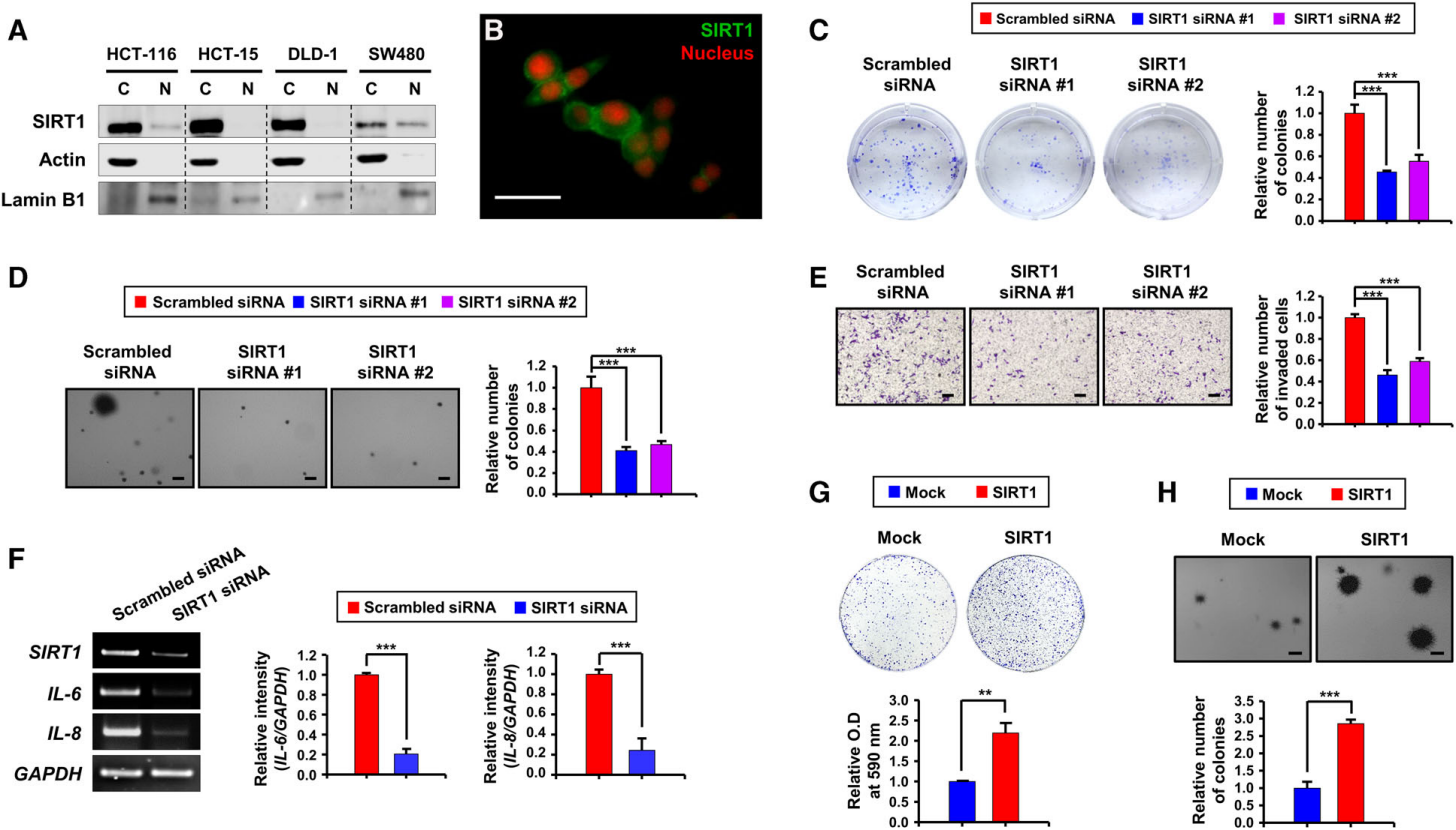
Effects of SIRT1 depletion or overexpression on colony formation and invasiveness of colon cancer cells. (A) Cytosolic and nuclear extracts from human colon cancer cell lines (HCT‐116, HCT‐15, DLD‐1, and SW480) were subjected to immunoblot analysis. Actin and lamin B1 were used as cytosolic and nuclear markers, respectively. C, cytosolic; N, nuclear. This experiment was conducted once. (B) Immunocytochemical analysis of SIRT1 in HCT‐116 cells. Nuclei were stained with propidium iodide (PI). Representative image was selected from the staining performed on three independent samples. Scale bar represents 200 μm. (C) HCT‐116 cells were transiently transfected for 48 h with scrambled or two different SIRT1 siRNA designed against different regions of the *SIRT1* gene, and clonogenic efficiency of cells was measured as described in Materials and methods. Differences in the means among the groups were assessed by one‐way ANOVA with Tukey's multiple comparisons test. The data are presented as the mean ± S.D. (*n* = 3 per group): ***P < 0.001. (D) Following transfection of HCT‐116 cells with scrambled or two different SIRT1 siRNA, the colony‐forming ability of cells was assessed by the anchorage‐independent growth assay. Each scale bar represents 200 µm. One‐way ANOVA with Tukey's multiple comparisons test was used to determine the significance. The values are presented as the mean ± S.D. (*n* = 3 per group): ***P < 0.001. (E) For the invasion assay, HCT‐116 cells transiently transfected with scrambled or two different SIRT1 siRNA were seeded into the Matrigel chambers. Cells invaded through the Matrigel‐coated membranes were stained with 0.05% crystal violet. Each scale bar represents 200 µm. One‐way ANOVA with Tukey's multiple comparisons test was adopted to measure the significance. The results are shown as the mean ± S.D. of three independent experiments: ***P < 0.001. (F) HCT‐116 cells were transiently transfected with scrambled or SIRT1 siRNA #1, and the mRNA levels of IL‐6 and 8 were determined by RT‐PCR analysis. Student's *t*‐test was employed to compare the means between two groups. The values are presented as the mean ± S.D. (*n* = 3 per group): ***P < 0.001. (G, H) SW480 cells were transiently transfected with mock or SIRT1 vector for 48 h. Cell growth was measured by both clonogenic (G) and anchorage‐independent growth (H) assays. Each scale bar represents 200 µm. Student's *t*‐test was used to determine the means between mock and SIRT1 vector. The data are presented as the mean ± S.D. (*n* = 3 per group): **P < 0.01, ***P < 0.001.

**Fig. 2:**
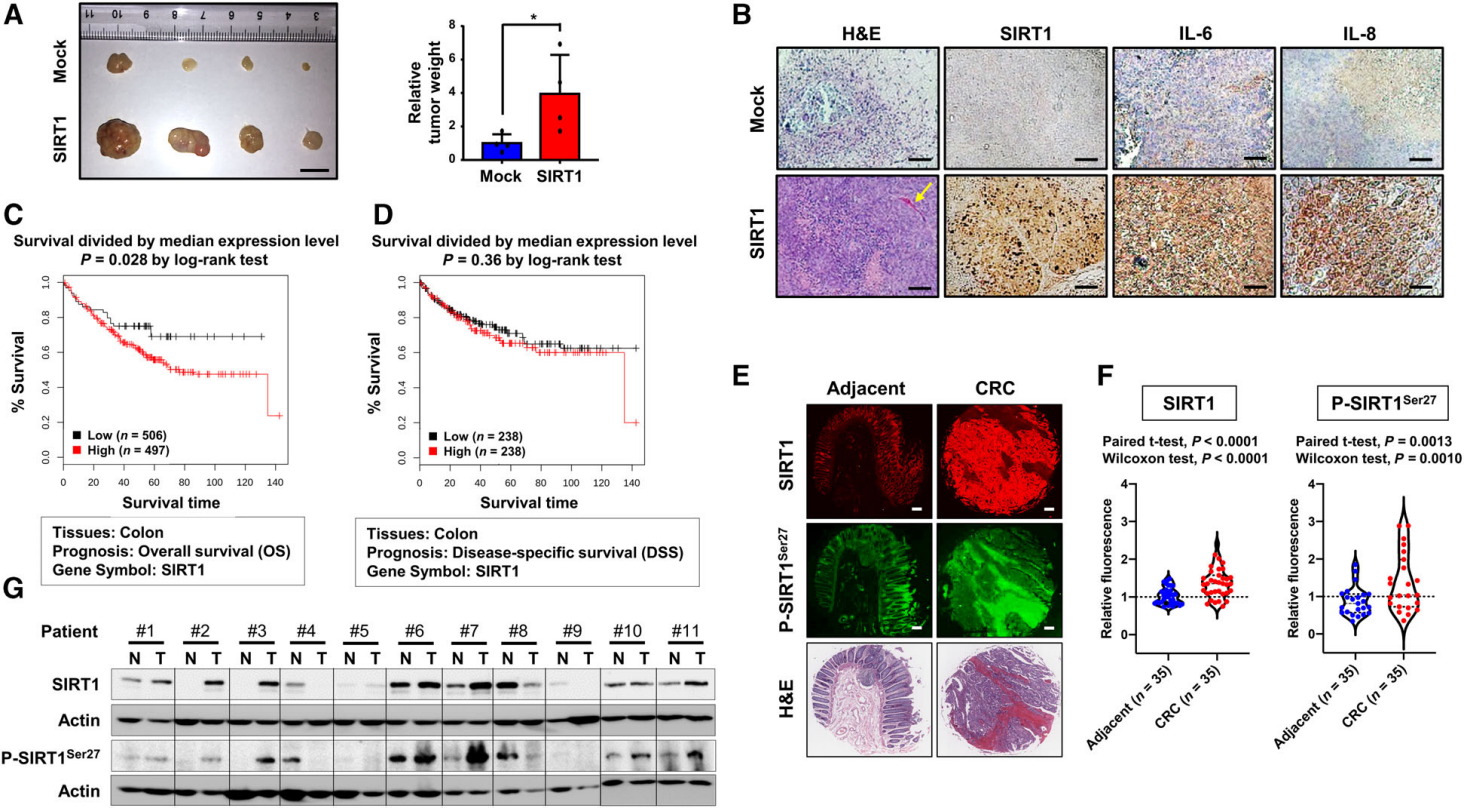
Overexpression of P‐SIRT1^Ser27^ in CRC tissues and its implications for SIRT1 stability and proliferation and migration of colon cancer cells. (A) SW480 cells (5 × 10^6^) stably expressing mock or SIRT1 vector were injected subcutaneously into the flank of BALB/c nude mice. Photographs show the xenograft tumors resected from mice at the time of harvest (day 50). The tumor volume and the tumor weight of the mice at the time of harvest were measured. Comparison of the means between mock and SIRT1 vector group was determined by Student's *t*‐test. The results are shown as the mean ± S.D. of four xenografts for each group: *P < 0.05. Scale bar represents 1 cm. (B) Histological structures in sections from excised xenograft tumors were analyzed by H&E staining (first column). The yellow arrow indicates neovascularization. The expression of SIRT1, IL‐6, and 8 in excised xenograft tumors were examined by immunohistochemical analysis (second to last columns). Each scale bar represents 100 µm. The data are presented as the mean ± S.D. of three independent samples for each group in Fig. S1H: *P < 0.05; **P < 0.01. (C) Kaplan–Meier survival curve of overall survival in colon cancer patients with high (*n* = 497) and low (*n* = 506) levels of SIRT1 based on the data collected from the GENT2 database (http://gent2.appex.kr). (D) Kaplan–Meier survival curve of disease‐specific survival in colon cancer patients with high (*n* = 238) and low (*n* = 238) levels of SIRT1 based on the data obtained from the GENT2 database (http://gent2.appex.kr) (E) Immunofluorescence staining of tissue microarray containing thirty‐five pairs of colon adenocarcinoma and matched adjacent normal colon tissues was conducted using anti‐SIRT1 and P‐SIRT1^Ser27^ antibodies. The adjacent normal area is 1.5 cm distant from the tumor, which was taken by a histologist. Images of H&E‐stained tissue sections were offered by US Biomax Inc. Each scale bar represents 200 µm. (F) The fluorescence intensity was measured by image analysis software ImageJ, and results are shown as a violin plot. Statistics were calculated using both paired *t*‐test and Wilcoxon matched‐pairs test. (G) The protein levels of SIRT1 and P‐SIRT1^Ser27^ in colon cancer and corresponding normal tissues were measured by Western blot analysis. The data are presented as the mean ± S.D. of eleven pairs of human tissue specimens in Fig. S2D: *P < 0.05.

**Fig. 3:**
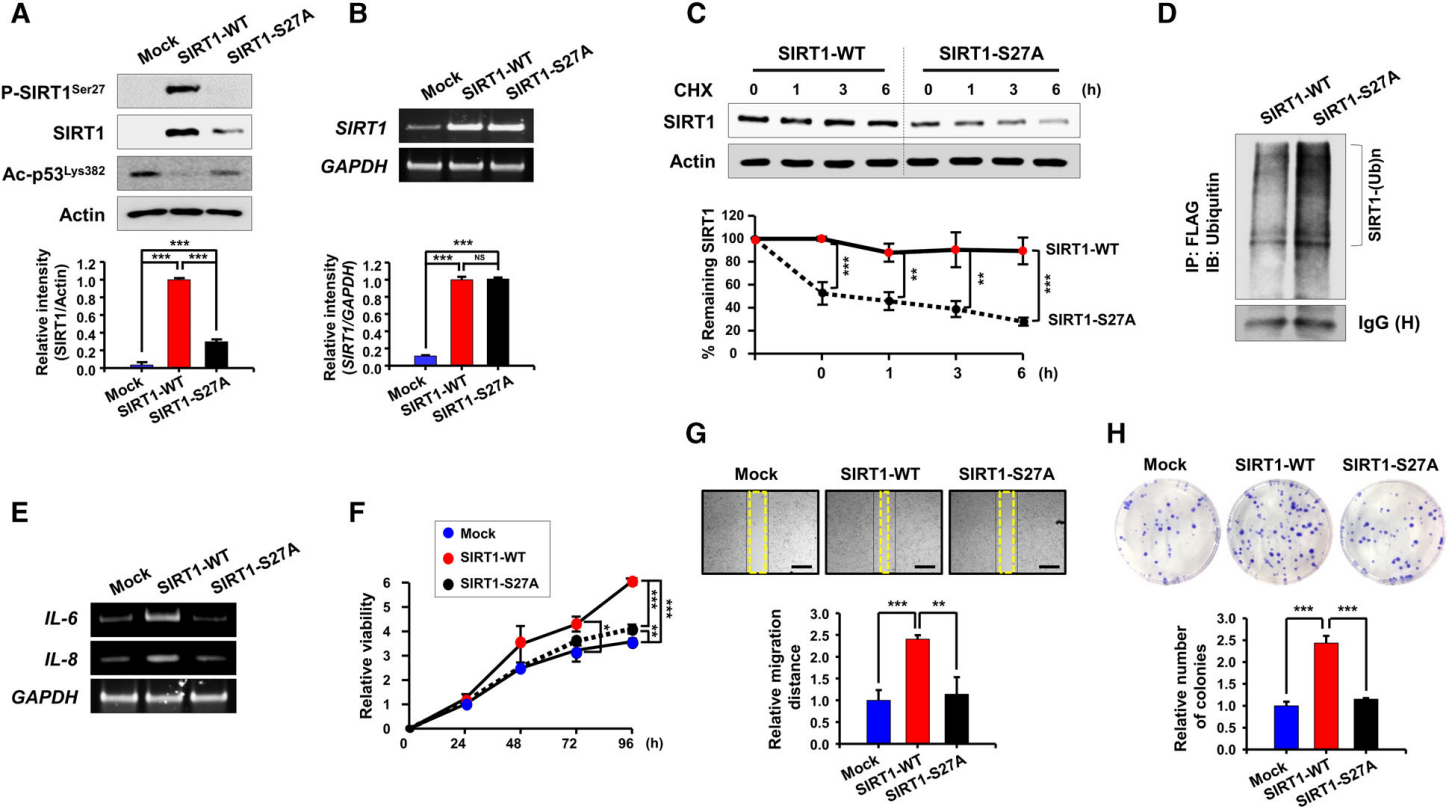
Comparison of the stability of SIRT1‐WT and SIRT1‐S27A and their effects on the viability, migration, and clonogenicity of HCT‐116 cells. (A, B) Following transfection of HCT‐116 cells with mock, SIRT1‐WT, or SIRT1‐S27A vector for 48 h, protein (A) and mRNA (B) levels of SIRT1 were measured by RT‐PCR and Western blot analyses, respectively. The level of p53 acetylated on lysine 383 (Ac‐p53^Lys382^) was used as an indicator of SIRT1 deacetylase activity. Differences in the means among the groups were determined by one‐way ANOVA with Tukey's multiple comparisons test. The results are presented as the mean ± S.D. (*n* = 3): ***P < 0.001. ns, not significant. (C) For the pulse‐chase analysis, HCT‐116 cells were incubated with CHX (20 μg·mL^−1^) for indicated time periods following transfection with SIRT1‐WT or SIRT1‐S27A. The protein level of SIRT1 remaining at each time point was determined by immunoblot analysis. Comparison of the means between two groups was determined by Student's *t*‐test. The data are presented as the mean ± S.D. (*n* = 3 per group): **P < 0.01; ***P < 0.001. (D) HCT‐116 cells were transiently transfected with FLAG‐tagged SIRT1‐WT or SIRT1‐S27A, followed by MG‐132 (20 μm) treatment for 2 h. Whole‐cell lysates were subjected to immunoprecipitation with an anti‐FLAG antibody, and Western blot analysis was performed using an antibody against ubiquitin. This experiment was carried out once. (E) Following transfection of HCT‐116 cells with the indicated plasmids, the mRNA levels of *IL‐6* and *IL‐8* were examined by RT‐PCR. This experiment was performed once. (F) For the cell viability assay, HCT‐116 cells were cultured for indicated time periods following transfection with the indicated plasmids. One‐way ANOVA with Tukey's multiple comparisons test was used to determine the significance. The results are shown as the mean ± S.D. of triplicates: *P < 0.05; **P < 0.01; ***P < 0.001. (G) HCT‐116 cells transfected with the indicated plasmids were subjected to the wound healing assay as described in Materials and Methods. Each scale bar represents 500 µm. Differences in the means among the groups were assessed by one‐way ANOVA with Tukey's multiple comparisons test. The data are presented as the mean ± S.D. of three independent experiments: **P < 0.01; ***P < 0.001. (H) HCT‐116 cells transfected with the indicated plasmids were seeded in 6‐well plates for the clonogenic assay. One‐way ANOVA with Tukey's multiple comparisons test was performed for the statistical comparison. The values are presented as the mean ± S.D. of three independent experiments: ***P < 0.001.

### 2.13 Xenograft assay

**Fig. 4:**
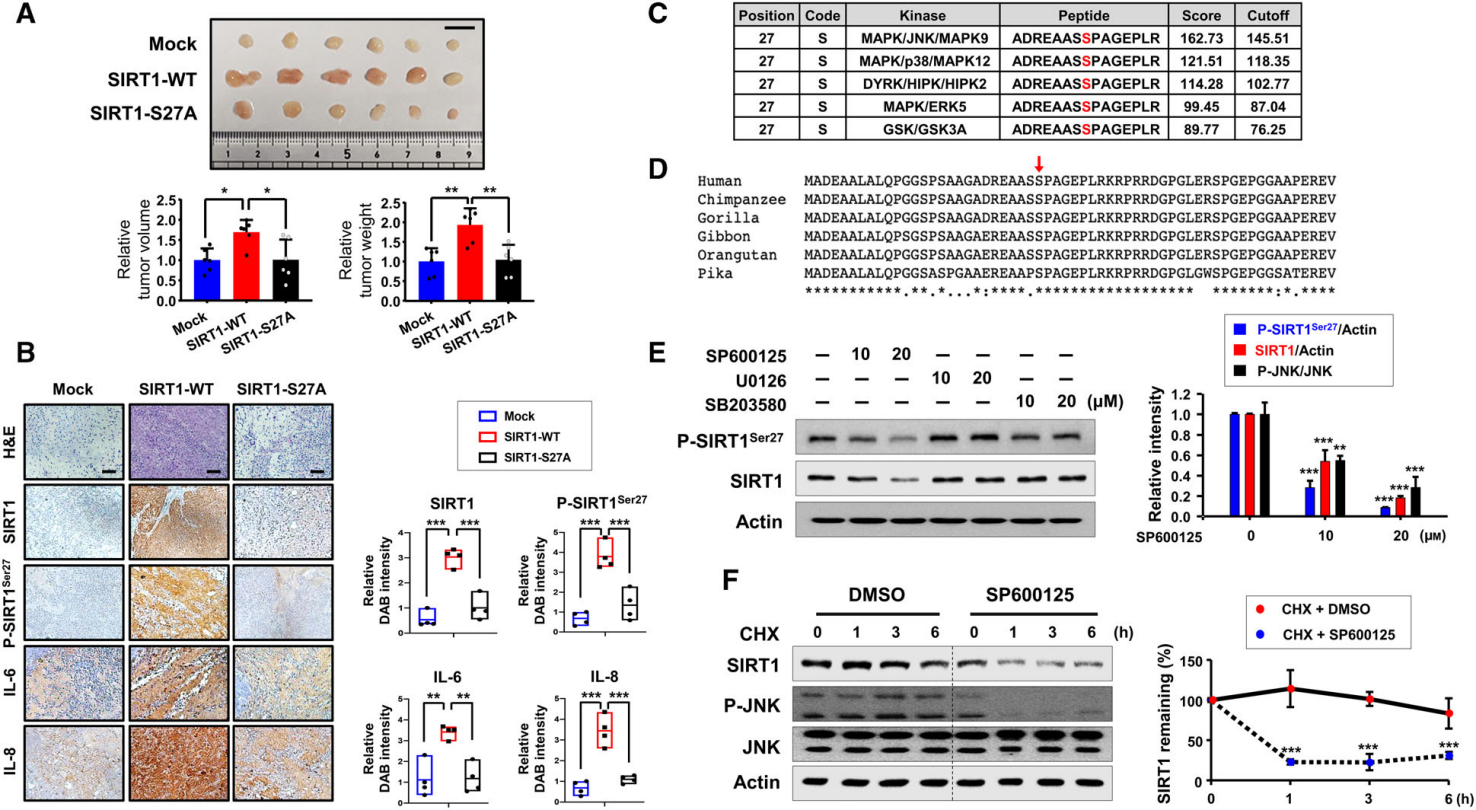
Comparison of the effects of SIRT1‐WT and SIRT1‐S27A on the tumorigenicity of HCT‐116 cells. (A) HCT‐116 cells (5 × 10^6^) transfected with mock, SIRT1‐WT, or SIRT1‐S27A vector were inoculated subcutaneously into the flank of BALB/c nude mice. Photographs show the xenograft tumors collected from mice at the end of the experiment (day 20). The tumor volume and the tumor weight of the mice at the time of harvest were measured, and the one‐way ANOVA with Tukey's multiple comparisons test was performed for the statistical comparison. The results are shown as the mean ± S.D. of six xenografts for each group: *P < 0.05; **P < 0.01. Scale bar represents 1 cm. (B) H&E staining and immunohistochemical analysis of SIRT1, P‐SIRT1^Ser27^, IL‐6, and IL‐8 were performed on sections from resected xenograft tumor. Each scale bar represents 100 µm. For quantification of each target, the DAB intensity was measured by image analysis software FIJI (the enhanced version of imagej2). Statistics were calculated using the one‐way ANOVA with Tukey's multiple comparisons test, and results are presented as the mean ± S.D. (*n* = 4 per group): **P < 0.01; ***P < 0.001. (C) Identification of high‐confidence kinase candidates contributing phosphorylation of SIRT1 at serine 27 by gps 5.0 (http://gps.biocuckoo.cn/). (D) Multiple sequence alignment using clustalw software (https://www.genome.jp/tools‐bin/clustalw) showing an evolutionarily conserved phosphorylation site of SIRT1 at serine 27 (arrow) between different species. The positions which have a fully conserved residue are indicated by asterisks. (E) HCT‐116 cells were treated with two different concentrations (10 and 20 µm) of a JNK inhibitor (SP600125), an ERK inhibitor (U0126) or a p38 inhibitor (SB203580) for 3 h. The protein levels of P‐SIRT1^Ser27^ and SIRT1 were measured by Western blot analysis. One‐way ANOVA with Tukey's multiple comparisons test was used to determine the significance. The results are shown as the mean ± S.D. (*n* = 3): **P < 0.01; ***P < 0.001. (F) HCT‐116 cells were treated with CHX (20 μg·mL^−1^) for indicated periods in the absence or presence of SP600125 (20 μm). Comparison of the means between two groups was determined by Student's *t*‐test. The values are presented as the mean ± S.D. of three independent experiments: ***P < 0.001.

Due to lack of thymus‐dependent immune function [35], BALB/c nude mice have been widely accepted as recipients of human tumor cell xenografts [36]. Four‐week‐old male BALB/c nude mice (weight 20 ± 1–2 g) supplied by RaonBio, Inc. (Yongin, South Korea) were housed in plastic cages in the specific pathogen‐free and temperature (22 ± 1 °C)‐ and humidity (40–60%)‐controlled facility with a 12‐h light/12‐h dark cycle. Following one week of acclimation, mice were divided into two and three groups for experiment in Fig. 2A,B and Fig. 4A,B, respectively. For the formation of xenograft tumors, 5 × 10^6^ SW480 cells stably expressing mock or SIRT1 re‐suspended in equal volumes of PBS and Matrigel (total volume of 200 μL) were subcutaneously injected into the flank of mice. For another experiment, 5 × 10^6^ HCT‐116 cells harboring mock or SIRT1‐WT or SIRT1‐S27A were prepared in the same way as delineated above and inoculated into the flank of mice. The tumors were monitored every 3 to 4 days. At the end of the experiment, mice were euthanized in a chamber by CO_2_ inhalation. After mice were sacrificed, xenograft tumors were resected and fixed in formalin for further investigation such as hematoxylin and eosin (H&E) staining and immunohistochemical (IHC) analysis. All procedures and protocols for animal experiments were reviewed and approved by the Institutional Animal Care and Use Committee (IACUC) of Seoul National University (authorization number: SNU‐190614‐2). In terms of the translational application, these animal models could be limited by the fact that the genetic background and histology of the xenograft tumors frequently do not reflect the respective human tumor.

### 2.14 Collection of human specimens

Colon tumor and matched adjacent normal colon tissues were acquired from eleven patients with stage I‐III colon cancer aged 19–89 years who were enrolled in an Institute’s tissue bank and underwent curative surgical resection in the period from January 2017 to December 2019 at Gachon University Gil Medical Center (Incheon, South Korea). Patients with multiple primary cancer and hereditary colorectal cancer were excluded. Specimens were frozen in liquid nitrogen and stored in a −80 °C freezer. The research use of tissue collections for the present study was approved by the Institutional Review Board (IRB) of Gachon University Gil Medical Center (authorization number: GDIRB2020‐040). The study methodologies conformed to the standards set by the Declaration of Helsinki, and the experiments were undertaken with the understanding and written consent of each subject.

### 2.15 Immunofluorescence analysis

Two human paraffin‐embedded colon cancer tissue array slides with matched adjacent normal colon tissue (US Biomax, Inc., cat. no. CO703; Rockville, MD, USA) were subjected to deparaffinization with xylene. Following antigen retrieval with heated citrate buffer, sections were permeabilized and blocked as described previously [8]. One slide was incubated with anti‐P‐SIRT1^Ser27^ antibody, and the other one was incubated with anti‐SIRT1 together with anti‐P‐SIRT1^Ser47^ antibodies for dual staining overnight at 4 °C. These slides were washed with PBS and labeled with FITC and FITC plus TRITC‐conjugated secondary antibodies for 1 h at room temperature, respectively. The slides were then analyzed under a fluorescence microscope.

### 2.16 IHC analysis

Cells were seeded on coverslips and cultured for 48 h. After blocking with 5% bovine serum albumin, cells were incubated with an antibody against Snail overnight at 4 °C. Cells were then washed with PBS and were labeled with TRITC‐conjugated secondary antibody for 1 h at room temperature. For visualization of the nuclei, cells were further stained with Hoechst solution (Invitrogen by Thermo Fisher Scientific Inc.; Waltham, MA, USA). The slides were then analyzed under a fluorescence microscope.

### 2.17 MTT assay

Cells (0.5 × 10^4^) were seeded in 48‐well plates. After incubation for the indicated periods of time, cells were treated with the MTT solution (final concentration; 1 mg·mL^−1^) for an additional 4 h. After supernatant withdrawal, DMSO was added (400 μL to each well) to dissolve the formazan crystals formed in intact cells. Two hundred μL of the resultant solution was transferred to a 96‐well plate, and the absorbance at 570 nm was read using a microplate reader.

### 2.18 Transcription factor activation profiling plate array

Nuclear extracts prepared from HCT‐116 cells expressing SIRT1‐WT or SIRT1‐S27A were subjected to the transcription factor activation profiling array (Signosis, Inc., cat. no. FA‐1002; Santa Clara, CA, USA) to monitor the activity of ninety‐six transcription factors simultaneously in one plate for a single sample following the manufacturer’s instructions. The activity‐dependent luminescent signal was detected as relative light units (RLUs) by using a microplate luminometer and was regarded significant when the changes in RLUs between SIRT1‐WT and SIRT1‐S27A were over twofold.

### 2.19 Statistical analysis

All statistical analyses were carried out using graphpad prism software (version 8.4.3). Comparison of the means between two groups was determined by Student's *t*‐test. For a comparison of matched samples from patients with colon cancer, paired *t*‐test and Wilcoxon matched‐pairs signed rank test were performed. Differences in the means among more than two groups were assessed by one‐way ANOVA with Tukey's multiple comparisons test. The data are presented as the mean ± standard deviation (S.D.) and considered statistically significant if *P* < 0.05.

## 3 Results

### 3.1 Silencing of SIRT1 attenuates the oncogenic capabilities of human colon cancer cells

We first investigated the role of SIRT1 in proliferation and growth of human colon cancer cells. SIRT1 is highly expressed in human colon cancer (e.g., HCT‐116) cells at the basal levels [8, 17, 37]. Notably, SIRT1 overexpression in HCT‐116 cells and other colon cancer cell lines was predominantly observed in the cytoplasm (Fig. 1A,B). Knockdown of SIRT1 resulted in decreased clonogenicity (Fig. 1C with the absolute values in Fig. S1A), anchorage‐independent growth (Fig. 1D with the absolute values in Fig. S1B), and invasiveness (Fig. 1E with the absolute values in Fig. S1C) of HCT‐116 cells.

It has been suggested that proinflammatory cytokines produced by tumor cells could stimulate the proliferation and invasion of tumor cells themselves, creating an autocrine loop [38, 39, 40, 41, 42]. Among them, IL‐6 and IL‐8 have been known to promote the growth and migration of colon cancer cells [43, 44]. We therefore examined whether SIRT1 could affect the production of these two cytokines in HCT‐116 cells. RT‐PCR analysis revealed that siRNA‐mediated depletion of SIRT1 led to a decline in expression of both cytokines at the transcriptional level (Fig. 1F). Furthermore, the mRNA levels of *c‐Myc*, *Bmi‐1*, and *Sox‐2,* which are considered to be oncogenic in the colon cancer, were reduced by SIRT1 siRNA (Fig. S1D).

### 3.2 Ectopic expression of SIRT1 augments proliferation of cultured human colon cancer cells and tumorigenicity *in vivo*

SW480 cells display a relatively lower level of endogenous SIRT1, compared to other colon cancer cells examined (Fig. S1E). When SW480 cells were transiently transfected with the SIRT1 vector, they became much more capable of forming colonies than mock‐transfected cells in clonogenic (Fig. 1G with the absolute values in Fig. S1F) and anchorage‐independent growth (Fig. 1H with the absolute values in Fig. S1G) assays. To further assess the role of SIRT1 in tumor formation *in vivo*, SW480 cells stably expressing SIRT1 were subcutaneously inoculated into nude mice. Stable ectopic expression of SIRT1 led to a marked increase in the volume and the weight of xenograft tumors compared with those of the control (Fig. 2A). In the postmortem analysis using excised tumors from mice, the H&E staining showed the histopathological features of xenograft tumor comprising cancerous cells densely along with a blood vessel (Fig. 2B). IHC analysis also exhibited a relatively intense brown staining of SIRT1, IL‐6, and IL‐8 in SIRT1‐overexpressing xenograft tumors relative to control tumors (Fig. 2B with the quantitative data in Fig. S1H). These findings further support that SIRT1 may act as a tumor promoter in colon cancer.

### 3.3 Both SIRT1 and P‐SIRT1^Ser27^ are aberrantly overexpressed in CRC

Notably, high expression levels of SIRT1 have a correlation with shorter overall survival (Fig. 2C) and disease‐specific survival (Fig. 2D) in colon cancer patients. The excessive phosphorylation of SIRT1 on serine 47 has been observed in tissue specimens from patients with CRC, implying its pathophysiological relevance to colorectal tumorigenesis [23]. However, SIRT1 is known to be phosphorylated on other serine residues, including serine 27 [17, 19]. We found that both SIRT1 and phosphorylated SIRT1 (P‐SIRT1^Ser27^) were prominently overexpressed in human colon tumor specimens compared with those in corresponding adjacent normal tissues (Fig. 2E with the quantitative data shown in Fig. 2F). Moreover, Spearman's correlation analysis indicated that P‐SIRT1^Ser27^ was positively correlated with the total level of SIRT1 (Fig. S2A). Other human colon tumor samples paired with matched normal counterparts, which showed substantial alterations in the histological structure (Fig. S2B), also showed that both SIRT1 and SIRT1 phosphorylated on Ser serine 27 were upregulated in tumor tissues in most cases (Fig. 2G with the quantitative data in Fig. S2C and the absolute values in Fig. S2D), and their relationship was highly positive with a correlation coefficient of 0.7511 (Fig. S2E). SIRT1 and P‐SIRT1^Ser27^ were barely detectable in some tumor specimens. Thus, 3 out of 11 cases displayed much lower levels of SIRT1 and P‐SIRT1^Ser27^ than the other tumor specimens. These three cases are at relatively lower stages such as 1 or 2, but a significant correlation was not established between the cancer stage and the degree of overexpression of SIRT1 or P‐SIRT1^Ser27^ (Fig. S2F with patient information in S2G). Similar observations were made in human colon cancer cell lines. Both P‐SIRT1^Ser27^ and total SIRT1 levels were highly elevated in three out of four human colon cancer cell lines relative to normal colon epithelial (CCD841CoN) cells (Fig. S1E).

### 3.4 Phosphorylation of SIRT1 on serine 27 enhances its stabilization

Phosphorylation of SIRT1 on the specific amino acid residues has been proposed to serve as regulatory mechanisms determining its protein stability or catalytic activity [17, 18, 19, 20, 21, 22]. To ascertain the effect of phosphorylation on serine 27 of SIRT1 on its stability and oncogenic potential, a phosphorylation‐defective mutant (SIRT1‐S27A) in which serine is substituted by alanine was employed. The SIRT1‐S27A mutant cells exhibited decreased protein expression of SIRT1, concomitantly with increased acetylation of p53 on lysine 382 (Fig. 3A). However, the mRNA expression of SIRT1 remained unchanged (Fig. 3B). In addition, SIRT1‐S27A mutant showed a shortened half‐life of SIRT1 compared to that of wild‐type SIRT1 (SIRT1‐WT) (Fig. 3C), which was accompanied by an increase in ubiquitination (Fig. 3D). These data suggest that stabilization of SIRT1 protein is attributable to its phosphorylation on serine 27.

We next examined the impact of SIRT1 phosphorylation on the oncogenicity of HCT‐116 cells. Serine to alanine substitution rendered HCT‐116 cells incompetent to express *IL‐6* and *IL‐8* as compared to SIRT1‐WT‐expressing cells (Fig. 3E). Concomitantly, SIRT1‐WT cells exhibit markedly enhanced cell viability while the viability of HCT‐116 cells expressing SIRT1‐S27A remained nearly the same as that of mock‐transfected cells (Fig. 3F). Moreover, HCT‐116 cells transfected with SIRT1‐WT showed increased abilities to migrate (Fig. 3G with the absolute values in Fig. S3A) and to form colonies (Fig. 3H with the absolute values in Fig. S3B) compared to mock‐transfected cells, but these capabilities were abolished in HCT‐116 cells transfected with SIRT1‐S27A.

### 3.5 Phosphorylation of SIRT1 on serine 27 enhances tumorigenicity of HCT‐116 cells *in vivo*

We next compared the tumorigenic potential of HCT‐116 cells carrying SIRT1‐WT or SIRT1‐S27A *in vivo*. When these cells were injected into the BALB/c nude mice, xenograft tumors derived from cells expressing SIRT1‐WT showed increases in the volume and the weight of tumors compared with those derived from mock‐transfected cells (Fig. 4A). In contrast, mice bearing xenograft tumors generated from cells harboring SIRT1‐S27A exhibited the tumor volume and the weight similar to those of mock control mice (Fig. 4A). In addition, xenograft tumors formed by SIRT1‐S27A‐transfected cells displayed less‐dense stroma visualized by H&E staining and diminished expression of SIRT1, IL‐6, and IL‐8 as well as P‐SIRT1^Ser27^ (Fig. 4B), indicative of the impaired function and stability of SIRT1 due to its defective phosphorylation.

It has been suggested that various kinases are involved in the phosphorylation of SIRT1. In particular, mitogen‐activated protein kinases (MAPKs) play a role in regulating the stability of SIRT1, positively or negatively [17, 18, 45]. Computational prediction of SIRT1 phosphorylation sites performed by Group‐based Prediction System (gps) 5.0 software indicates that serine 27 of SIRT1 is eligible for phosphorylation. Notably, all three arms of the MAPK cascade (JNK, ERK, and p38) are ranked within the top five candidate kinase hits responsible for phosphorylation of SIRT1 at serine 27 among the CMGC group of protein kinases including cyclin‐dependent kinases, MAPKs, glycogen synthase kinases, and CDK‐like kinases (Fig. 4C). In addition, multiple sequence alignment showed that serine 27 of SIRT1 is highly conserved across various mammalian species (Fig. 4D). To clarify which kinase(s) is/are responsible for serine 27 phosphorylation of SIRT1, HCT‐116 cells were treated with the SP600125, U0126, and SB203580, which are chemical inhibitors of JNK, ERK, and p38 MAPK, respectively. Of these, only SP600125 significantly reduced the expression levels of P‐SIRT1^Ser27^ and SIRT1 (Fig. 4E). These results suggest that JNK appears to be a putative kinase that catalyzes the SIRT1 phosphorylation on serine 27.

Next, the association between JNK‐dependent phosphorylation and stabilization of SIRT1 was investigated. Treatment of HCT‐116 cells with SP600125 did not alter the mRNA level of SIRT1 as evaluated by RT‐PCR (Fig. S4A) and qPCR analyses (Fig. S4B). Curcumin has been known to induce ubiquitin‐dependent degradation of SIRT1 [8]. Like curcumin, treatment with SP600125 provoked ubiquitination of SIRT1 (Fig. S4C), thereby decreasing the half‐life of SIRT1 as assessed by the CHX chase assay (Fig. 4F). These findings suggest that JNK‐dependent phosphorylation of SIRT1 contributes to the stabilization of SIRT1, promoting the growth and progression of colon cancer.

### 3.6 SIRT1 binds Snail and positively regulates its transcriptional activity

**Fig. 5:**
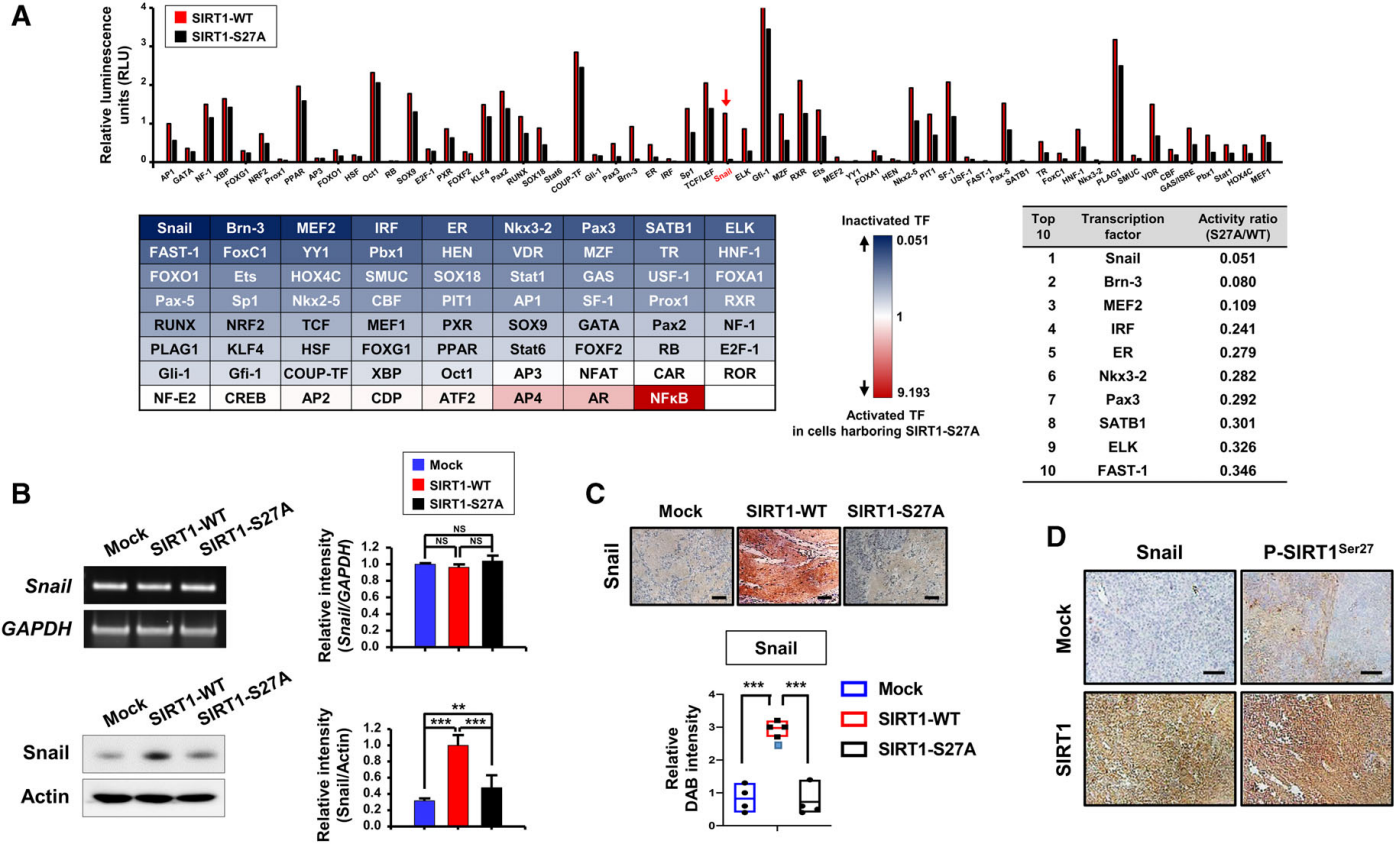
Strong inactivation of Snail in HCT‐116 cells expressing SIRT1‐S27A. (A) Nuclear extracts prepared from HCT‐116 cells transfected with SIRT1‐WT or SIRT1‐S27A were subjected to the transcription factor activation profiling array to analyze the activity of ninety‐six different transcription factors. The top 10 hits are listed in the Table in an ascending order of the SIRT1‐S27A to SIRT1‐WT ratio. The red arrow denotes the top hit whose activity was most reduced in HCT‐116 cells harboring the SIRT1‐S27A mutation. (B) Following transfection of HCT‐116 cells with mock, SIRT1‐WT, or SIRT1‐S27A vector for 48 h, the mRNA (upper panels) and protein (lower panels) levels of Snail were measured by RT‐PCR and immunoblotting analyses, respectively. Differences in the means among the groups were assessed by one‐way ANOVA with Tukey's multiple comparisons test. Data are presented as the mean ± S.D. (*n* = 3 per group): **P < 0.01; ***P < 0.001. NS, not significant. (C) Immunohistochemical (IHC) analysis of Snail was carried out on sections from resected xenograft tumor corresponding to the Fig. 4A. Each scale bar represents 100 µm. The DAB intensity was assessed by image analysis software fiji (the enhanced version of imagej2). Statistics were calculated using one‐way ANOVA with Tukey's multiple comparisons test, and the data are presented as the mean ± S.D. (*n* = 4 per group): ***P < 0.001. (D) IHC analysis of Snail and P‐SIRT1^Ser27^ was performed on sections from resected xenograft tumor corresponding to the Fig. 2B. Each scale bar represents 100 µm. The results are shown as the mean ± S.D. of three independent samples for each group in Fig. S5B: *P < 0.05; **P < 0.01.

Since SIRT1 positively regulates expression of *IL‐6* and *IL‐8* (Fig. 1F and Fig. 3E) that play crucial roles in proliferation of colon cancer cells, we speculate that SIRT1 may partly promote the colon cancer cell growth and migration through production of these cytokines. However, it remained unanswered how SIRT1 could upregulate the transcription of genes encoding IL‐6 and IL‐8. Because SIRT1 is mainly localized in the cytosol of human colon cancer cell lines (Fig. 1A,B), it is unlikely that SIRT1 acts as a transcriptional coactivator to enhance the expression of *IL‐6* and *IL‐8* or induces histone deacetylation to epigenetically silence the genes that are involved in inhibition of their transcription. This prompted us to investigate an intermediate factor that could cooperate with SIRT1 for the production of IL‐6 and IL‐8. The results of transcription factor profiling revealed that Snail, a zinc‐finger transcription factor, is the top hit showing decreased activity (Fig. 5A), and nuclear factor‐κB (NF‐κB) was the top hit showing increased activity (Fig. S5A) in HCT‐116 cells harboring SIRT1‐S27A relative to HCT‐116 cells expressing SIRT1‐WT. However, NF‐κB has been well known to be responsible for transcription of *IL‐6* and *IL‐8* [46, 47, 48] and was hence excluded from the list of qualified candidates.

We then measured the expression level of Snail. As shown in Fig. 5B, the protein level of Snail decreased in HCT‐116 cells transfected with SIRT1‐S27A compared with SIRT1‐WT cells without any change in mRNA expression. IHC analysis shows that Snail is strongly expressed in xenograft tumors derived from HCT‐116 cells carrying SIRT1‐WT, relative to those from SIRT1‐S27A mutant cells (Fig. 5C). Notably, P‐SIRT1^Ser27^ expression was highly elevated in xenograft tumors formed by SIRT1 overexpressing SW480 cells as well (Fig. 5D with the quantitative data in Fig. S5B). In line with this notion, robust induction of Snail1 in SIRT1 overexpressing cells was abrogated by the JNK inhibitor SP600125, while the effect of the pharmacologic inhibition of JNK was not much prominent in SIRT1‐S27A expressing cells (Fig. S6).

### 3.7 SIRT1 contributes to deacetylation and translocation of Snail to regulate the production of IL‐6 and IL‐8

**Fig. 6:**
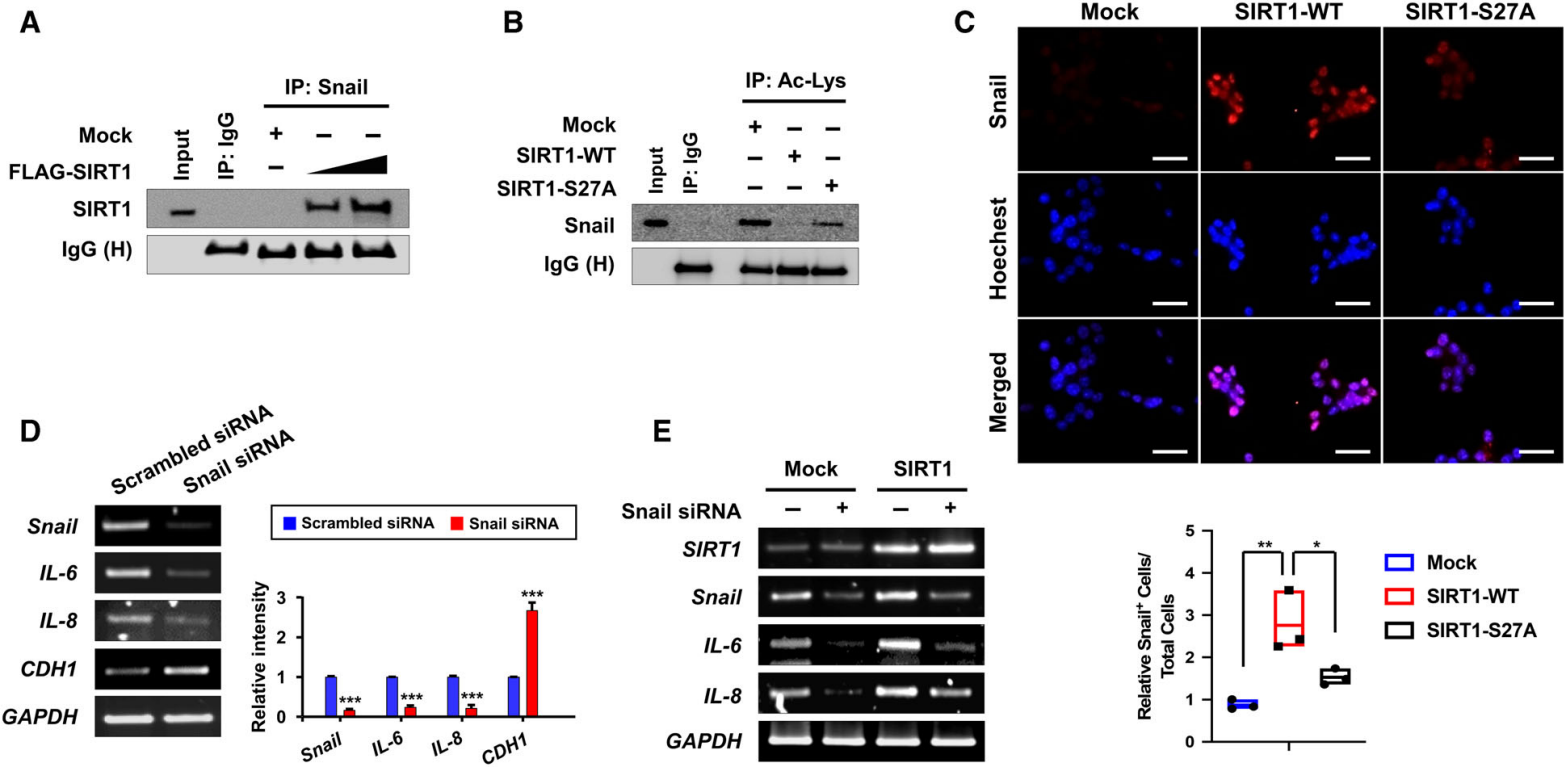
Cooperation SIRT1 and Snail in the regulation of IL‐6 and 8 production. (A) Following transfection of HCT‐116 cells with mock or SIRT1 vector (3 or 6 μg), whole‐cell lysates were subjected to immunoprecipitation with an anti‐Snail antibody, and Western blot analysis was performed using an anti‐SIRT1 antibody. This experiment was conducted once. (B) HCT‐116 cells were transiently transfected with mock, SIRT1‐WT, or SIRT1‐S27A for 48 h. Whole‐cell lysates were subjected to immunoprecipitation with an anti‐acetylated lysine antibody, and immunoblot analysis was performed using an antibody against Snail. This experiment was carried out once. (C) After the transfection of HCT‐116 cells with the indicated plasmids, immunocytochemical analysis was performed using an anti‐Snail antibody. Nuclei were stained with Hoechst 33342. The intensities of TRITC and Hoechst staining were assessed by image analysis software FIJI (the enhanced version of ImageJ2) to quantify Snail as % of positive nuclei. Statistics were calculated using one‐way ANOVA with Tukey's multiple comparisons test, and the data are presented as the mean ± S.D. of three independent experiments: *P < 0.05; **P < 0.01. The scale bar represents 200 μm. (D) HCT‐116 cells were transiently transfected with scrambled or Snail siRNA for 48 h. The mRNA levels of Snail, IL‐6, 8, and E‐cadherin were measured by RT‐PCR analysis. Student's *t*‐test was used to determine the means between two groups. The results are shown as the mean ± S.D. (*n* = 3 per group): ***P < 0.001. (E) Following co‐transfection of HCT‐116 cells with mock or SIRT1 vector and scrambled or Snail siRNA, the mRNA levels of SIRT1, Snail, IL‐6, and IL‐8 were assessed by RT‐PCR analysis. This experiment was performed once.

Consideraing that SIRT1 has deacetylase activity, we examined whether SIRT1 could affect the acetylation status of Snail. We found that ectopically expressed SIRT1 physically interacted with Snail (Fig. 6A). Further, ectopic expression of SIRT1 resulted in deacetylation of Snail while serine 27 to alanine substitution abolished this effect (Fig. 6B). Since Snail is a transcription factor, nuclear localization should be essential for its transcriptional activity. Notably, Snail was less detectable in the nucleus of HCT‐116 cells harboring SIRT1‐S27A compared with HCT‐116 cells expressing SIRT1‐WT (Fig. 6C). These findings suggest that SIRT1‐mediated deacetylation of Snail1 may facilitate the nuclear transportation of this transcription factor. To validate the possibility that Snail may be involved in SIRT1‐mediated production of IL‐6 and IL‐8, we checked whether Snail could positively regulate the expression/production of these cytokines. Snail is known to be a strong repressor of E‐cadherin transcription. Ablation of Snail by siRNA led to a decline in the levels of both *IL‐6* and *IL‐8* as well as restoration of E‐cadherin gene (*CDH1*) expression (Fig. 6D). Furthermore, overexpression of SIRT1 enhanced the transcription of *IL‐6* and *IL‐8* in HCT‐116 cells, which was impeded by knockdown of Snail (Fig. 6E). These results suggest that Snail could mediate SIRT1‐induced production of IL‐6 and IL‐8.

**Fig. 7:**
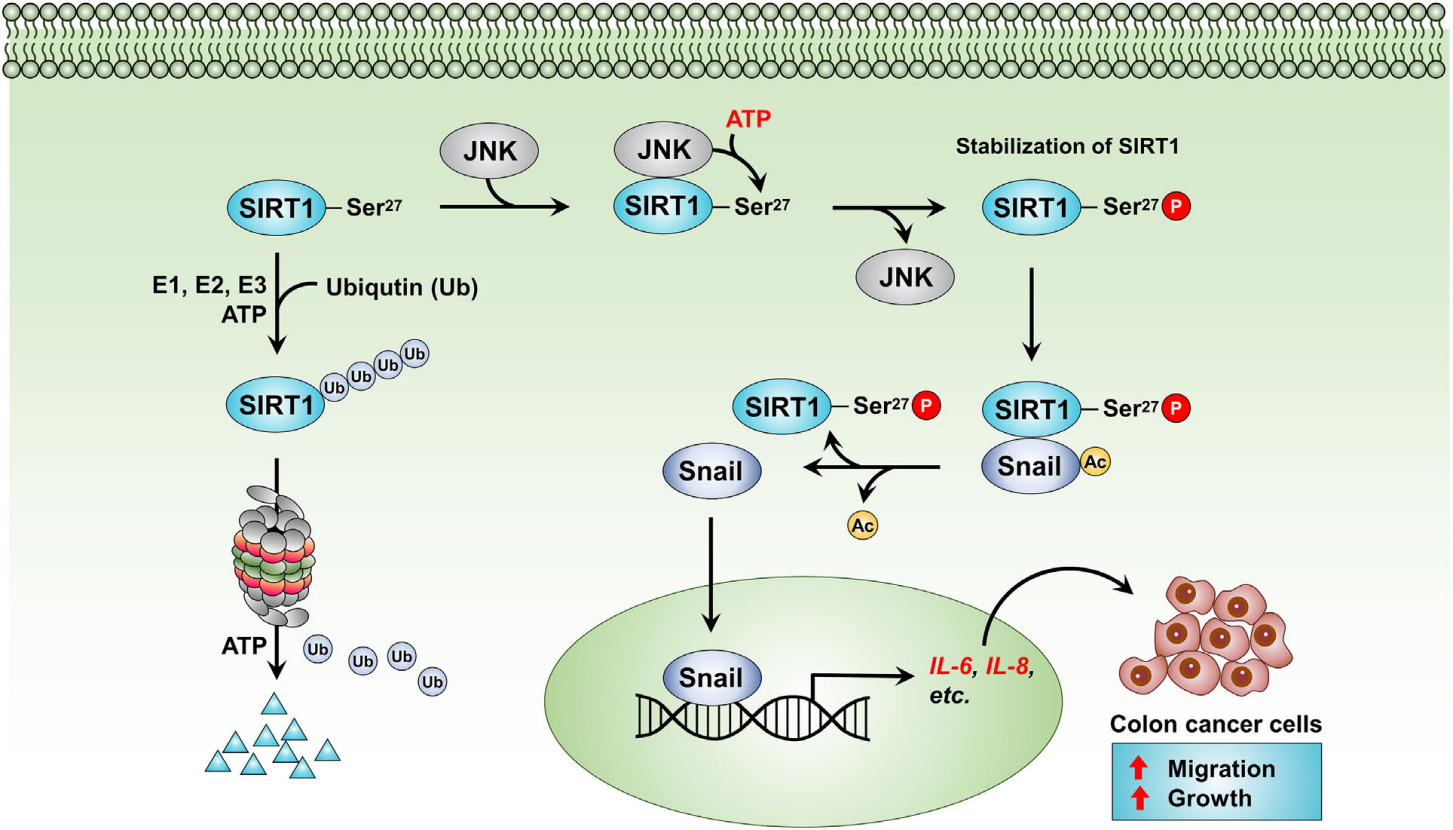
A proposed mechanism underlying contribution of aberrantly stabilized SIRT1 to oncogenicity of human colon cancer cells. JNK‐dependent phosphorylation of SIRT1 at Ser27 contributes to its stabilization and deacetylation of Snail to enhance the production of IL‐6 and IL‐8, thereby promoting proliferation and migration of human colon cancer cells.

## 4 Discussion

As one of the most universal post‐translational modifications, phosphorylation of proteins affects their biological activity, stability, subcellular localization, binding affinity to other proteins, etc. To date, SIRT1 has been known to contain more than 20 phosphorylation sites [20, 21, 22, 49, 50, 51, 52, 53]. It has been reported that the levels of P‐SIRT1^Ser47^ as well as SIRT1 are higher in colorectal cancer tissues than in adjacent normal tissues [23]. Of note, P‐SIRT1^Ser47^ positively correlated with a cellular proliferation marker, Ki67 [23]. Thus, phosphorylation of SIRT1 at the serine 47 residue has been considered to have a distinctive role in the colon cancer progression. According to our tissue microarray analysis, however, the protein level of P‐SIRT1^Ser47^ was not prominently high in tumor tissues relative to the normal adjacent area from the same patient (data not shown), whereas phosphorylation on serine 27 was markedly elevated compared to the normal counterpart.

It has been suggested that serine 27 of SIRT1 is phosphorylated by JNK2, resulting in the enhanced protein stability in HCT‐116 cells [17]. Moreover, SIRT1 is phosphorylated at both serine 27 and 47 residues and stabilized by Ca^2+^/calmodulin‐dependent protein kinase β in human umbilical vein endothelial cells under pulsatile shear stress [19]. Contrary to the above observation, phosphorylation of mouse SIRT1 on serine 46, which corresponds to serine 47 of human SIRT1, is mediated by JNK1 and related to its ubiquitin‐dependent degradation [18]. Moreover, phosphorylation occurring on other serine or threonine residues is involved in catalytic activity rather than protein stability of SIRT1 [20, 21, 22, 53, 54].

In the current study, serine 27 to alanine substitution rendered SIRT1 susceptible to degradation, suggesting that phosphorylation on this amino acid is essential for SIRT1 protein stabilization. However, the mechanism by which phosphorylation of SIRT1 on Ser27 prevents its ubiquitination‐dependent proteasomal degradation remains to be further explored. Several reports have indicated that phosphorylation is a prerequisite for some proteins to interact with other proteins [55, 56]. Notably, Janus kinase 1‐mediated phosphorylation of SIRT1 is required for its interaction with the transcription factor STAT3 [51]. Phosphorylation of SIRT1 may facilitate the interaction between SIRT1 and another binding partner such as USP22, an endogenous stabilizer of SIRT1 acting as a specific deubiquitinase, to keep SIRT1 from ubiquitination. Alternatively, a phosphorylation‐induced conformational change of SIRT1 could block access of E3 ubiquitin ligase(s), thereby maintaining its stability.

Snail primarily serves as a transcriptional repressor in EMT, by regulating transcription of epithelial marker genes, including encoding E‐cadherin [26, 27, 28], Mucin‐1 [57], claudins [58], and occluding [59]. Some studies have focused on the role of Snail in the production of cytokines that affect the behavior of tumor cells and their neighboring cells in the tumor microenvironment. It has been reported that Snail upregulates proinflammatory genes, such as *IL‐1*α, *IL‐1*β, *IL‐*6, *IL‐*8, *CXCL1*, and *COX‐2*, in human oral keratinocytes [60]. In another study, ectopically overexpressed Snail upregulated the expression of IL‐8 by directly binding to the E3/E4 E‐boxes present in its gene promoter, thereby inducing self‐renewal activity of human colon cancer cell lines (SW480 and HCT15) [31].

A complementary DNA microarray showed that the immune response genes represent the most prominently upregulated group in the head and neck cancer cell line (FaDu) stably expressing Snail [30]. In particular, *CCL2* and *CCL5* were found to be the major downstream targets of Snail. According to this study, increased levels of these proinflammatory chemokines promoted the M2‐like polarization and recruitment of tumor‐associated macrophages, facilitating tumor progression. Interestingly, acetylation of Snail by CREB‐binding protein at lysine 146 and 187 residues was associated with inhibition of the repressor complex formation and consequently stabilization of Snail without affecting its DNA‐binding ability or subcellular localization [30]. On the other hand, our study revealed that SIRT1 deacetylated Snail by direct binding and promoted translocation of Snail into the nucleus, leading to upregulation of *IL‐6* and *IL‐8*. We speculate that SIRT1 drives Snail to promote colon cancer progression, not by provoking a conventional EMT‐related transcriptional repressor function but by inducing transcriptional activator activity of Snail. In this context, our study proposes a unique collaborative relationship between SIRT1 and Snail in the colon cancer growth and progression.

## 5 Conclusions

Phosphorylation of SIRT1 on the serine 27 residue by JNK induces its stabilization and subsequently deacetylation‐dependent nuclear translocation of Snail (Fig. 7). This leads to the transcription of proinflammatory cytokines IL‐6 and IL‐8, thereby stimulating migration and growth of human colon cancer cells.

## Conflict of interest

The authors declare no conflict of interest.

### Peer Review

The peer review history for this article is available at https://publons.com/publon/10.1002/1878‐0261.13143.

## Authors’ contributions

YHL, NYS, and YJS defined the research theme and designed the experimental approach. YHL and XF carried out the experiments. YHL, SJK, XF, DHK, JS, and HKN analyzed the data and interpreted the results. YHL and YJS wrote the manuscript. JS and SJK provided technical support. KOK and JHB supplied the human tissue specimens. All authors read and approved the final manuscript.

## Supporting information


**Fig. S1**. Assessment of oncogenic effects of SIRT1.
**Fig. S2**. Clinical relevance of SIRT1 and P‐SIRT1^Ser27^ overexpression to colon cancer development and progression.
**Fig. S3**. The absolute values of data on the effects of non‐phosphorylatable mutation of Ser27 on migration and clonogenicity of human colon cancer cells.
**Fig. S4**. Effects of pharmacologic inhibition of JNK on mRNA expression and ubiquitination of SIRT.
**Fig. S5**. Association between P‐SIRT1^Ser27^ and Snail.
**Fig. S6**. Comparative effects of JNK inhibition on SIRT1 phosphorylation and Snail expression in mock control, SIRT1‐WT, and SIRT1‐S27A cells.

## Data Availability

Data are contained within the article or Supporting information.
