# Supplementary material for: JNK‐mediated Ser27 phosphorylation and stabilization of SIRT1 promote growth and progression of colon cancer through deacetylation‐dependent activation of Snail
Source: Mol Oncol. 2022 Jan 4;16(7):1555–71. doi: 10.1002/1878-0261.13143 (PMC8978515; doi:10.1002/1878-0261.13143)
Supplement: Supplementary file 1 — Fig. S1. Assessment of oncogenic effects of SIRT1. Fig. S2. Clinical relevance of SIRT1 and P‐SIRT1Ser27 overexpression to colon cancer development and progression. Fig. S3. The absolute values of data on the effects of non‐phosphorylatable mutation of Ser27 on migration and clonogenicity of human colon cancer cells. Fig. S4. Effects of pharmacologic inhibition of JNK on mRNA expression and ubiquitination of SIRT. Fig. S5. Association between P‐SIRT1Ser27 and Snail. Fig. S6. Comparative effects of JNK inhibition on SIRT1 phosphorylation and Snail expression in mock control, SIRT1‐WT, and SIRT1‐S27A cells. [file MOL2-16-1555-s001.pdf]

A

	Scrambled siRNA	SIRT1 siRNA #1	SIRT1 siRNA #2
Biological Replicate 1	96	38	62
Biological Replicate 2	118	45	60
Biological Replicate 3	101	41	47

Data point: Cells were grown for 14 days

B

	Scrambled siRNA	SIRT1 siRNA #1	SIRT1 siRNA #2
Biological Replicate 1	83	35	45
Biological Replicate 2	70	33	39
Biological Replicate 3	83	29	35

Data point:Cells were incubated in semi-solid medium for 14 days

C

	Scrambled siRNA	SIRT1 siRNA #1	SIRT1 siRNA #2
Biological Replicate 1	2806	1280	1595
Biological Replicate 2	2837	1471	1729
Biological Replicate 3	2973	1221	1752

Data point: Cells were incubated for 48 hours

D

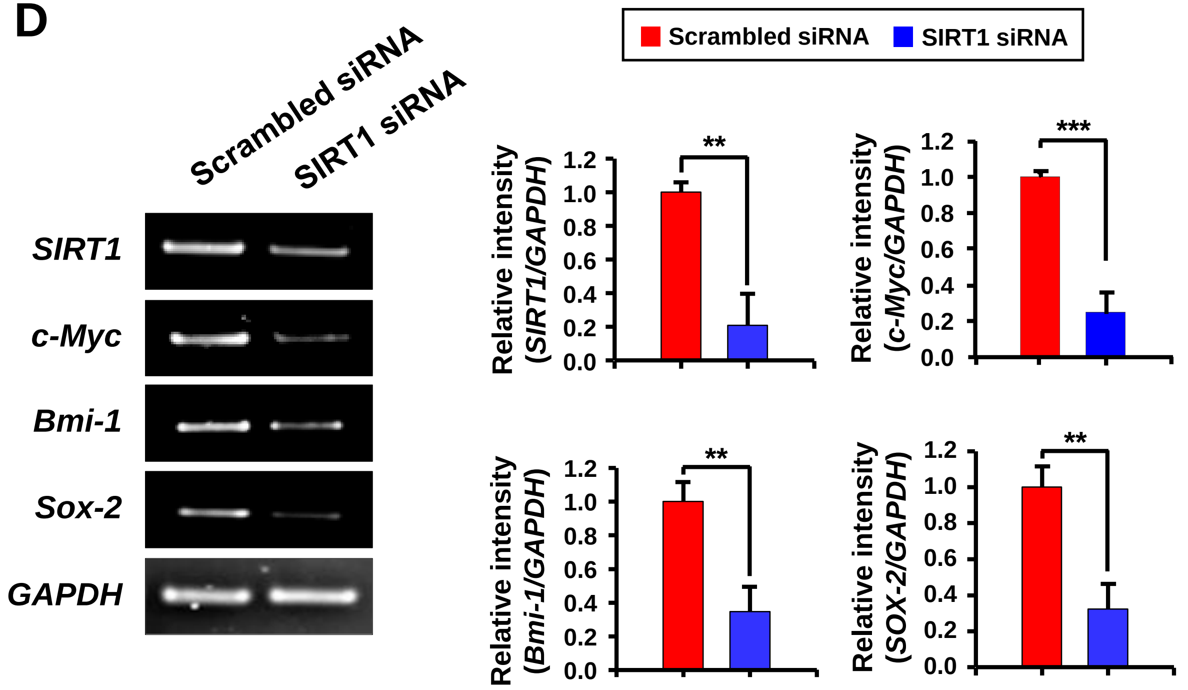

Supplementary Fig. S1

E

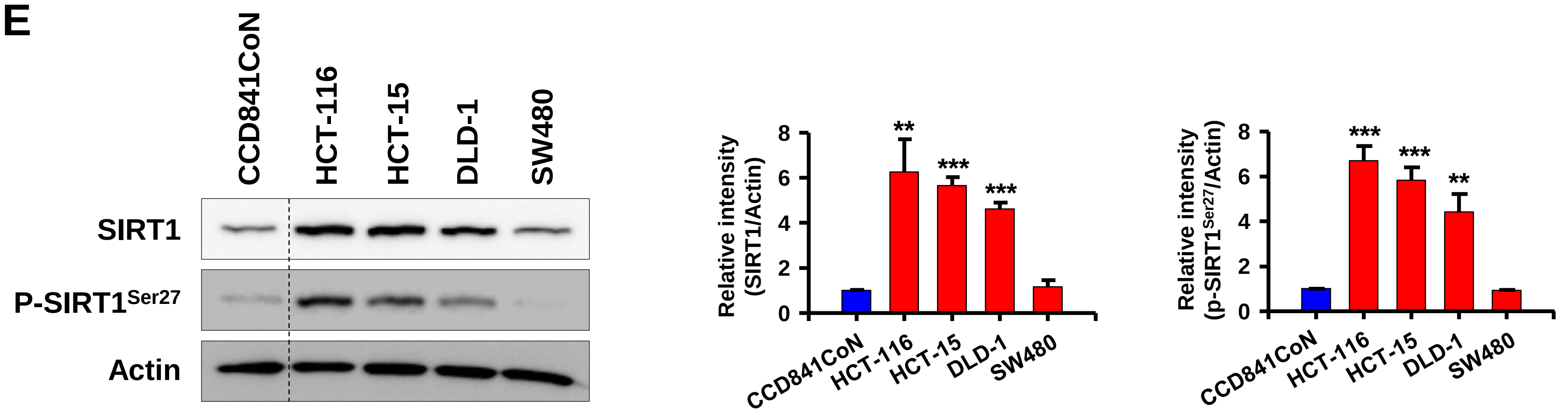

F

	Mock	SIRT1
Biological Replicate 1	0.108	0.223
Biological Replicate 2	0.11	0.222
Biological Replicate 3	0.107	0.268

Data point: Cells were grown for 14 days

G

	Mock	SIRT1
Biological Replicate 1	11	33
Biological Replicate 2	14	34
Biological Replicate 3	10	32

Data point: Cells were incubated in semi-solid medium for 14 days

H

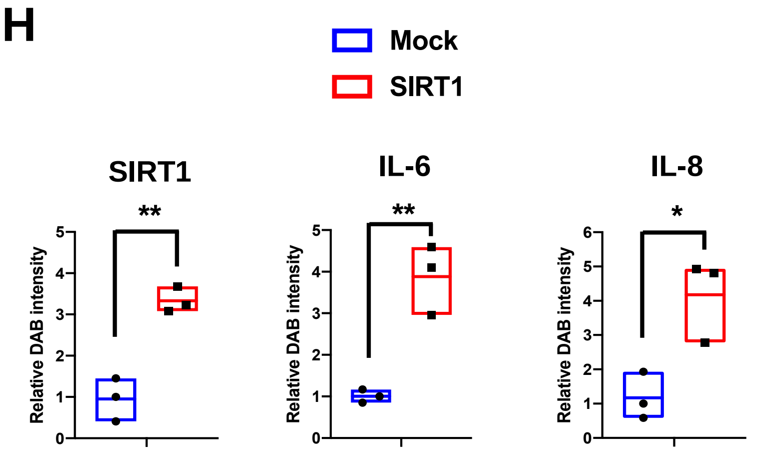

Supplementary Fig. S1 (continued)

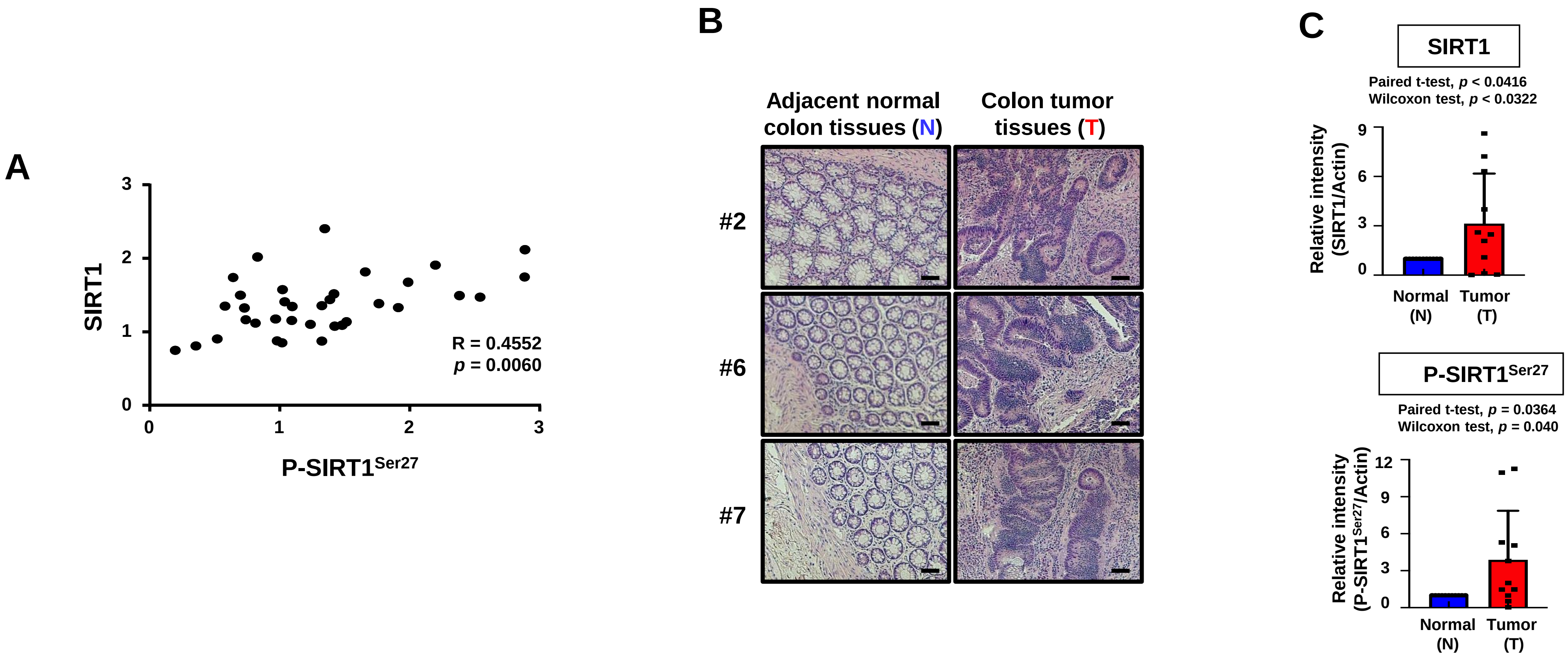

D

Patient #	1		2		3		4		5		6		7		8		9		10		11	
	N	T	N	T	N	T	N	T	N	T	N	T	N	T	N	T	N	T	N	T	N	T
SIRT1	1.00	2.60	0.18	1.53	0.21	1.32	0.57	0.02	0.05	0.10	5.79	14.33	1.47	10.62	5.78	0.57	0.17	0.00	1.00	1.08	0.62	2.47
P-SIRT1^{Ser27}	1.00	1.46	0.24	1.26	0.11	1.19	1.89	0.08	0.16	0.32	14.73	21.50	3.54	39.81	15.72	8.12	0.29	0.28	1.00	3.79	0.89	4.49

Supplementary Fig. S2

E

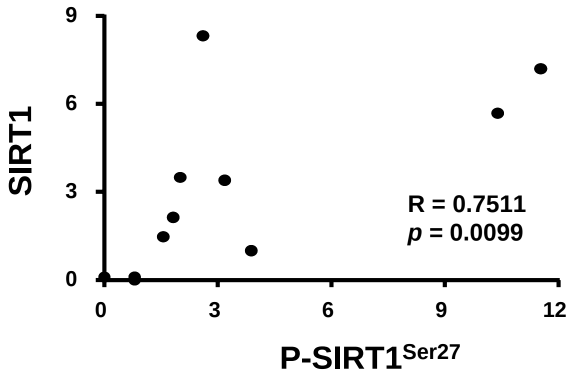

F

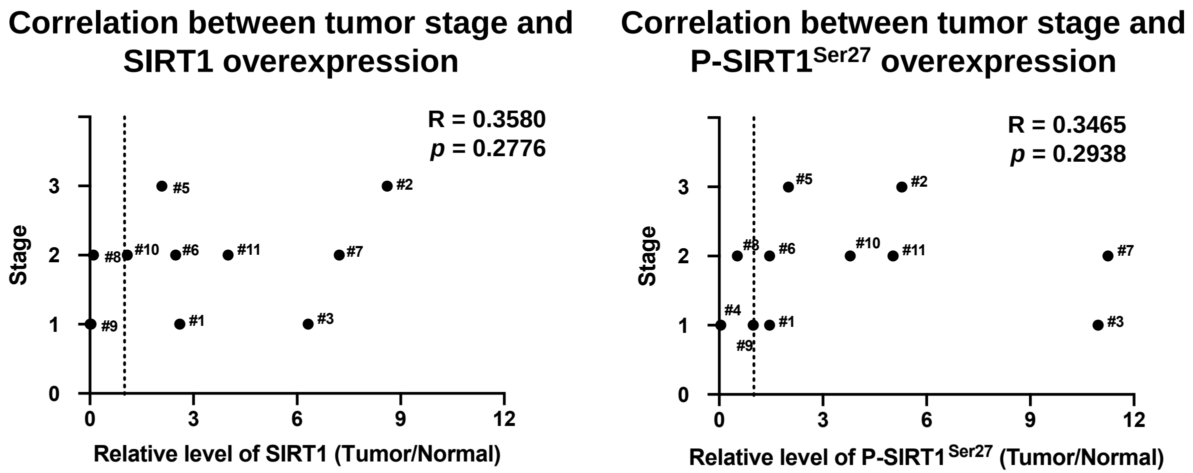

G

Patient #	Gender	Age	K-ras mutation	N-ras mutation	EGFR	MSI status	Differentiation type	Stage
1	F	54	WT	WT	Positive	MSS	Moderate	1
2	M	78	WT	WT	Positive	MSS	Well differentiated	3
3	M	63	Mut	WT	Positive	MSS	Moderate	1
4	F	64	Mut	WT	Positive	MSS	Moderate	1
5	M	71	WT	WT	Positive	MSS	Moderate	3
6	M	77	WT	WT	Positive	MSI-L	Moderate	2
7	F	79	WT	WT	Positive	MSS	Moderate	2
8	F	55	WT	WT	Positive	MSS	Moderate	2
9	M	73	Mut	WT	Positive	MSS	Moderate	1
10	F	70	WT	WT	Positive	MSS	Moderate	2
11	F	74	WT	WT	Positive	MSS	Moderate	2

Supplementary Fig. S2 (continued)

A

	Mock	SIRT1 -WT	SIRT1 -S27A
Biological Replicate 1	91.5	260.5	100.9
Biological Replicate 2	105.6	274.6	110.3
Biological Replicate 3	133.8	260.5	166.6

Data point: Cells were grown for 48 hours

B

	Mock	SIRT1 -WT	SIRT1 -S27A
Biological Replicate 1	48	100	51
Biological Replicate 2	39	115	49
Biological Replicate 3	44	104	51

Data point: Cells were incubated for 14 days

Supplementary Fig. S3

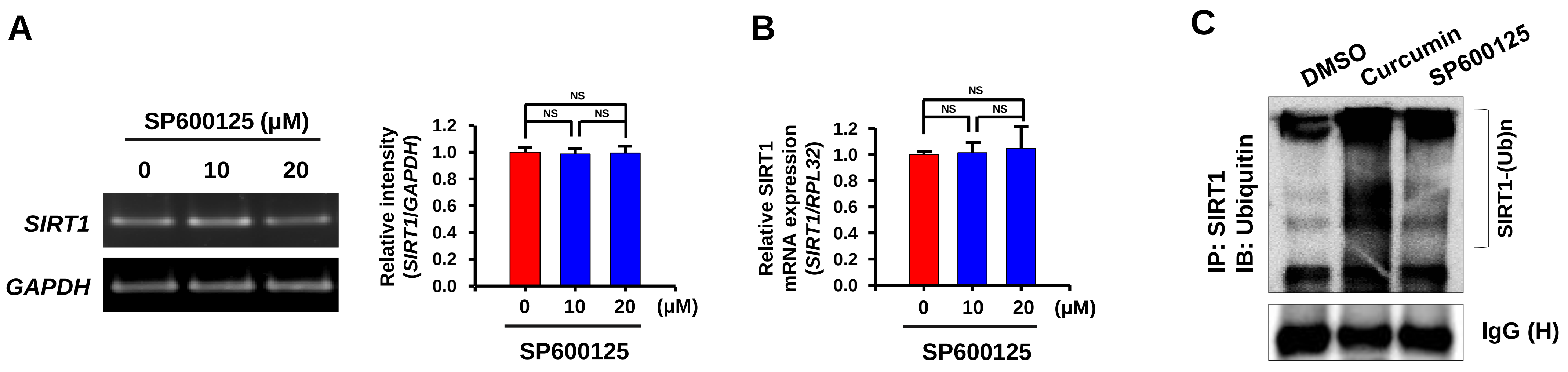

Supplementary Fig. S4

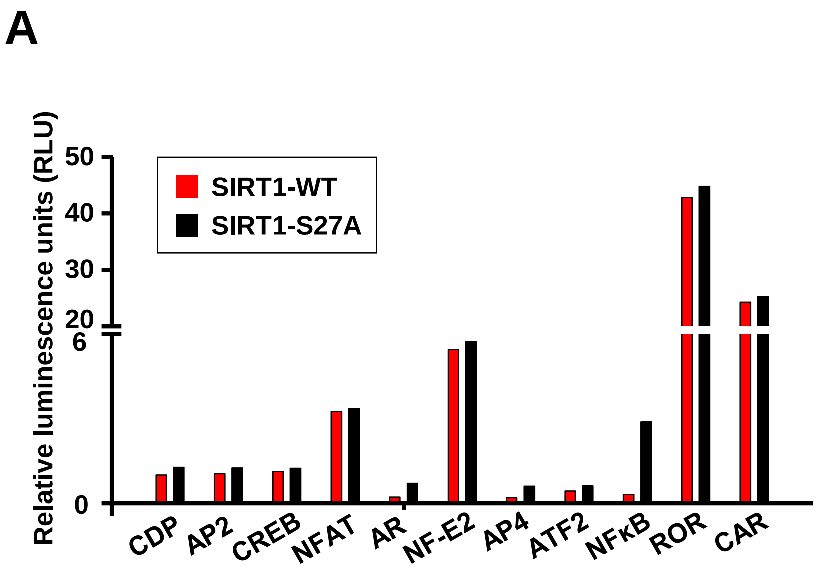

	Transcription factor	Activity ratio (S27A/WT)
1	NF-κB	9.193
2	AR	3.238
3	AP4	3.145

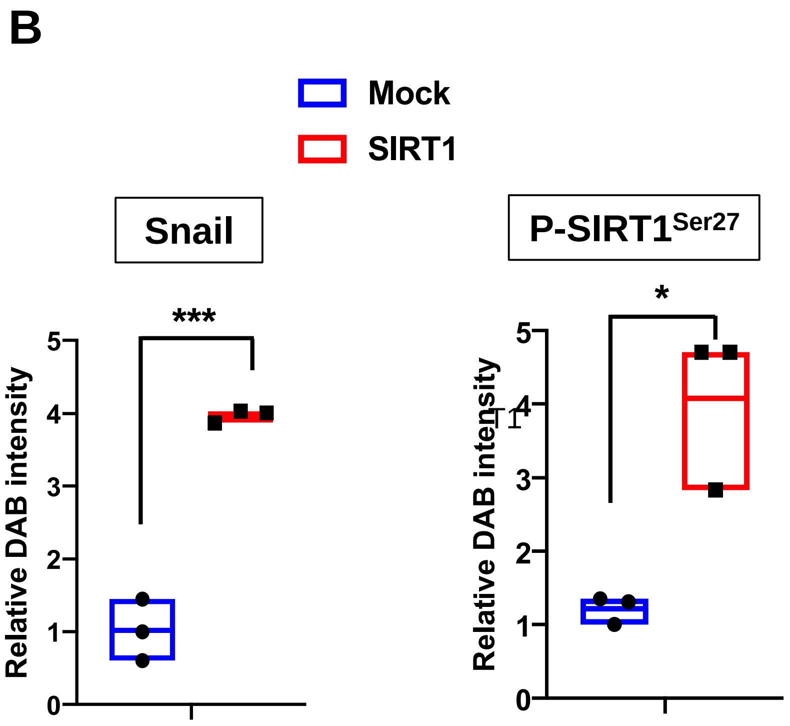

Supplementary Fig. S5

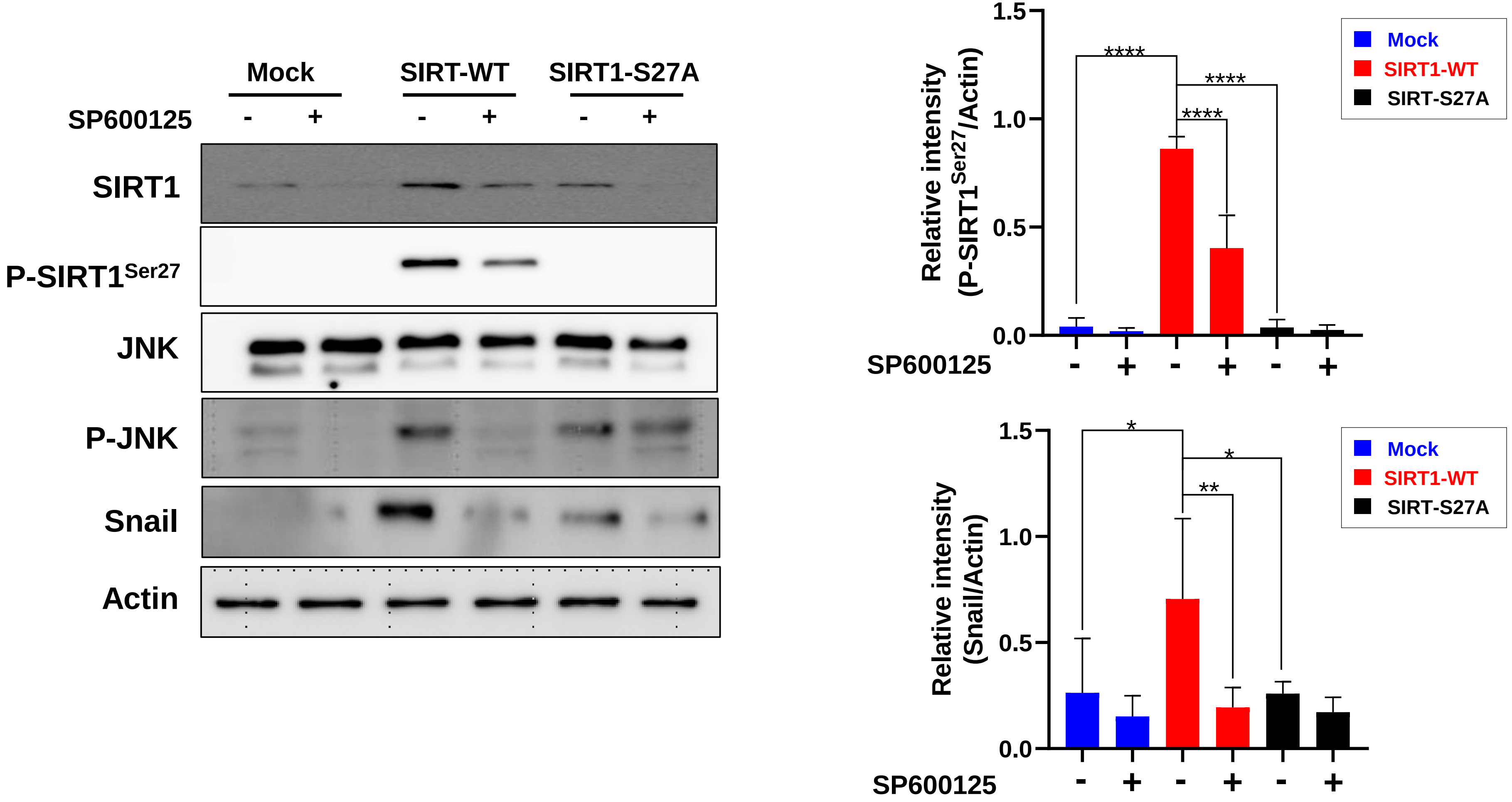

Supplementary Fig. S6
